# Chiral Self-Assembly of Porphyrins Induced by Chiral Carbon Dots

**DOI:** 10.3389/fchem.2020.00670

**Published:** 2020-08-07

**Authors:** Xiaowei Liu, Jiayi Lu, Jingqi Chen, Mengtian Zhang, Yingying Chen, Feifei Xing, Lingyan Feng

**Affiliations:** ^1^Materials Genome Institute, Shanghai University, Shanghai, China; ^2^College of Qianweichang, Shanghai University, Shanghai, China; ^3^College of Chemistry and Materials Science, Nanjing Normal University, Nanjing, China; ^4^College of Science, Department of Chemistry, Shanghai University, Shanghai, China

**Keywords:** chiral carbon dots, porphyrin, chiral templates, self-assembly, chirality transfer

## Abstract

Chirality plays a key role in many fields ranging from life to natural sciences. For a long time, chiral materials have been developed and used to interact with chiral environments. In recent years, fluorescent carbon dots (CDots) are a new class of carbon nanomaterials exhibit excellent optical properties, good biocompatibility, excellent water solubility, and low cost. However, chirality transfer between semiconductor CDots and organics remains a challenge. Herein, a facile one-step hydrothermal method was used to synthesize chiral CDs from cysteine (cys). The obtained chiral CDots can act as chiral templates to induce porphyrins to form chiral supramolecular assemblies. The successful transmission of chiral information provides more options for the development of various chiral composite materials and the preservation of chiral information in the future.

## Introduction

Chirality is one of the most interesting phenomena in nature, which means that an object doesn't overlap its mirror image (Wang et al., 2013). The most important biological macromolecules in life, such as sugars, protein, and nucleic acids, are all chiral (Milton et al., 2016). Therefore, chirality is widely concerned in scientific research, including drug recognition (Zhou et al., 2018), physiological activities (Wei et al., 2014), material preparation (Chen et al., 2019), enantioselective reactions (Yutthalekha et al., 2016), and pharmaceutical science (Yeom et al., 2020). Nanomaterials have broad application prospects in biosensors and biomedical fields due to their surface effect and small size effect (Albanese et al., 2012). Because the most important chiral biomacromolecules, such as protein and nucleic acid, are nanoscale, the research of chiral nanomaterials has also aroused peoples' interests.

Early chiral nanostructures were synthesized using organic structures as templates (Qiu et al., 2009). Inorganic materials can be adsorbed or deposited on top of the template and then removed by high temperature annealing. In addition, chiral molecular stabilizers such as amino acids have been successfully used in the synthesis of chiral quantum dots (Moloney et al., 2007). Up to now, many achievements have been made in the research of chiral nanomaterials. Among the chiral nanomaterials, chiral noble metal nanomaterials (Xu et al., 2019), and chiral semiconductor nanomaterials (Nakashima et al., 2009) have been gradually developed in recent years. However, the toxicity and high cost of metals limit their large-scale commercialization. Therefore, it is extremely urgent to prepare and develop new chiral nanomaterials.

As one of the most abundant elements in nature, carbon is widely distributed in the atmosphere and the earth's crust. Carbon-based nanomaterials such as carbon nanotubes (De Volder et al., 2013), nano-diamonds (Novoselov et al., 2005), and graphene (Geim and Novoselov, 2007) etc., play an increasingly important role in human society. As a new star in the family of carbon-based nanomaterials, fluorescent carbon dots (CDots) have attracted more and more attention in recent years (Lim et al., 2015). Compared with traditional semiconductor quantum dots and organic dyes, CDots not only maintain the advantages of low toxicity and good biocompatibility of carbon materials, but also have the advantages of good light stability, easy functionalization, low price, and easy large-scale synthesis (Baker and Baker, 2010), that can be applied in optical devices (Tian et al., 2017), biosensors (Feng et al., 2013), drug delivery (Gong et al., 2019), and optical imaging (Han et al., 2019).

Surprisingly, the combination of chirality and luminescent properties makes chiral-CDots have remarkable properties. Studies on chiral carbon dots show that L-CDots and D-CDots have different biological effects and applications. For example, L-CDots showed enhanced glycolysis in cells, while D-CDots had no similar effect, suggesting that chiral carbon dots can selectively controlled the energy metabolism of cells (Li F. et al., 2018). Chiral CDots also have the ability to promote the photosynthesis of mung bean plants (Zhang et al., 2018). In addition, L-CDots synthesized from L-Lysine can significantly remodeled the secondary structure of amyloid beta-42(Aβ-42) and inhibit the cytotoxicity, its fibril morphology, the aggregation process and membrane interaction of Aβ-42 (Malishev et al., 2018). Kang et al. reported that chiral CDots [(+)-D-CDots or (–)-L-CDots] could be prepared by electrochemical polymerization for 2–6 days from L- or D-glutamic acid in aqueous alkali, and the chirality of CDots could be regulated and even reversed by controlling electrolysis time. Moreover, the synthesized chiral carbon dots can modify maltose to different degrees to control the hydrolysis rate of glucose (Zhang et al., 2019). Recently, Yang and co-workers reported that the chiral carbon dots derived from cysteine can mimic topoisomerase I, which can selectively mediate the topological rearrangement of supercoiled DNA. Compared with L-CDots, D-CDots can more effectively catalyze the topological transformation of plasmid DNA from supercoiled structure to open-circular construction (Li et al., 2020). To sum up, biological effects are closely related to chiral properties of material, chiral carbon dots will have broader application prospects in many fields.

Porphyrins are natural tetrapyrrole ring macromolecules formed by the interaction of four pyrrole subunits α-carbon atoms with a methyl-bridge (=CH−). The special macrocyclic structure of porphyrins determines that porphyrins and their derivatives have good electron buffering, photoelectric magnetism and excellent chemical photostability. Porphyrins have been widely used in material (Tanaka and Osuka, 2015), molecular recognition (Ding et al., 2015), and catalysts (Maeda et al., 2015) due to their special macrocyclic planar conjugated structures. Porphyrin complexes were obtained by using the porphyrin rings with the properties of π-conjugation as monomers. Recently, Qu et al. reported that CDots can be used as electron donor and transporter, and porphyrins can be assembled on CDots through electrostatic and π-stacking interactions to form porphyrin-CDots supramolecular composites (Wang et al., 2011). The porphyrin is connected to CDots by electrostatic interaction to form the conjugate of CDot-TMPyP_4_, which can be used as a photosensitizer for photodynamic treatment of cancer (PDT) and has an effective therapeutic effect (Wang et al., 2014). Due to the high solubility of CDots and its ability as a good electron acceptor and transporter, porphyrin-CDots may open up potential applications for the development of these novel supramolecular systems in collector photovoltaic systems and photodynamic therapy devices.

**Figure 1:**
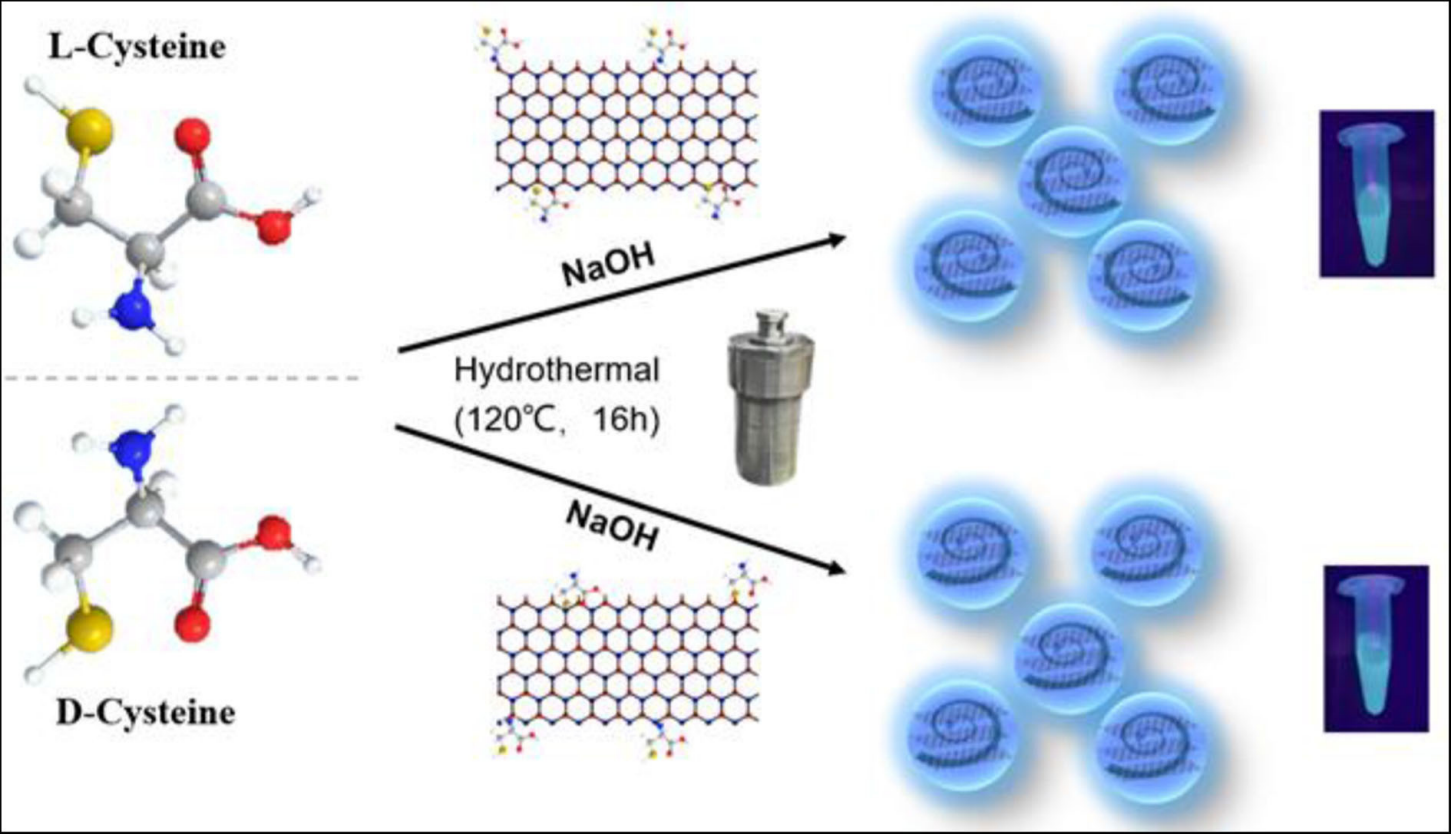
Design and synthesis of chiral carbon dots.

At present, the chirality transfer between semiconductor CDots and organics remains a challenge. It is also difficult to synthesize organic-inorganic chiral materials based on CDots. Due to the important role of porphyrins in many fields, it is expected that porphyrins can be assembled into chiral nanostructures in chiral environments. Herein, we prepared chiral CDots with a facile one-step hydrothermal method (Figure 1). The chiral CDots can act as chiral templates to induce organic porphyrins to form supramolecular assemblies. The successful transmission of chiral information provides more options for the development of various chiral composite materials and the preservation of chiral information in the future.

## Experimental Section

### Materials

L-cysteine (L-Cys), D-cysteine (D-Cys), and TMPyP_4_ (5, 10, 15, 20-Tetrakis (1-methyl-4-pyridinio) porphyrin tetra (p-toluenesulfonate) were purchased from Sigma-Aldrich (Shanghai, China). NaOH was obtained from Sinopharm Chemical Reagent Company (Shanghai, China). H_2_TPPS Hydrate (=Tetraphenyl porphyrin Tetrasulfonic Acid Hydrate) Ultra-high sensitive spectrophotometric reagent for transition metals were bought from Tokyo Chemical Industry (Shanghai, China). Dialysis tubes with molecular weight cut-off 1 KDa were obtained from Sangon Biotech (Shanghai, China). All the chemicals were analytical grade and used without further treatment. All solutions were prepared using ultrapure water (18.2 MΩ·cm) from a Milli-Q automatic ultrapure water system.

### Preparation of L/D-Cys-CDots

The chiral CDots were synthesized by a hydrothermal method (Hu et al., 2017). Typically, 0.6 g L- or D-Cys and 1 g NaOH were sufficiently dissolved in 10 mL water under ultrasonication for 30 min. Then, the above mentioned solution was transferred to a 25 mL Teflon-sealed autoclave and heated at 120°C for 16 h, giving rise to a brown sample. To remove impurities, the raw solution was dialyzed against a 1 KDa cellulose dialysis membrane for 3 days. Then aqueous solution was lyophilized to give a brownish solid.

### Chiral Supramolecular Assemblies

Porphyrin stock solution was prepared by dissolving porphyrin solid in ultrapure water under pH = 7 and the H_2_TPPS porphyrin concentrations were calculated using the molar extinction coefficient at 413 nm in water of 48,000 M^−1^ cm^−1^. The TMPyP_4_ porphyrin concentrations were also calculated the molar extinction coefficient at 422 nm in water of 26,000 M^−1^ cm^−1^. The L(D)-CDots+H_2_TPPS solution was prepared by mixing the chiral carbon dots (150 μg/mL) and H_2_TPPS solution (100 μM) at pH = 6.5. After incubation for 30 min, the pH dropped to 2.5 and porphyrin aggregation was induced. The L(D)-CDots+TMPyP_4_ solution was prepared by incubating TMPyP_4_ (100 μM) with chiral carbon dots (150 μg/mL) at pH = 7.0 for 24 h. To avoid undesired photochemical reactions and experimental errors, all steps have been done to avoid light.

### Characterization

Transmission electron microscopy (TEM) and high-resolution TEM images were obtained using FEI/Philips Tecnai F20 (200 kv) transmission electron microscopy. X-ray photoelectron spectroscopy (XPS) data were obtained using the Kratos Axis Ultra instrument (Manchester, UK) under a very high vacuum (<10^−8^ Torr). Fourier transform infrared (FT-IR) spectroscopy was obtained by Nicolet iS50, a Thermo Fisher company. UV-Vis absorption spectra were acquired on a Perkin-Elmer Lambda 750 UV-Vis spectrophotometer, and the slit width detail was 2.0 nm. Luminescent spectra were determined on Edinburgh instrument FS5 spectrofluorometer (Livingston, UK) (All the spectra were recorded at room temperature using 1 cm path-length cuvettes.) Electronic Circular Dichroism. CD spectra were recorded using Jasco J-810 at room temperature. A quartz cuvette with a 1 cm path length was used for all CD experiments. Conditions were as follows: scanning rate 50 nm/min, data pitch 0.2 nm, and D.I.T 2 s. Each CD spectrum was an average of at least three scans.

## Results and Discussion

**Figure 2:**
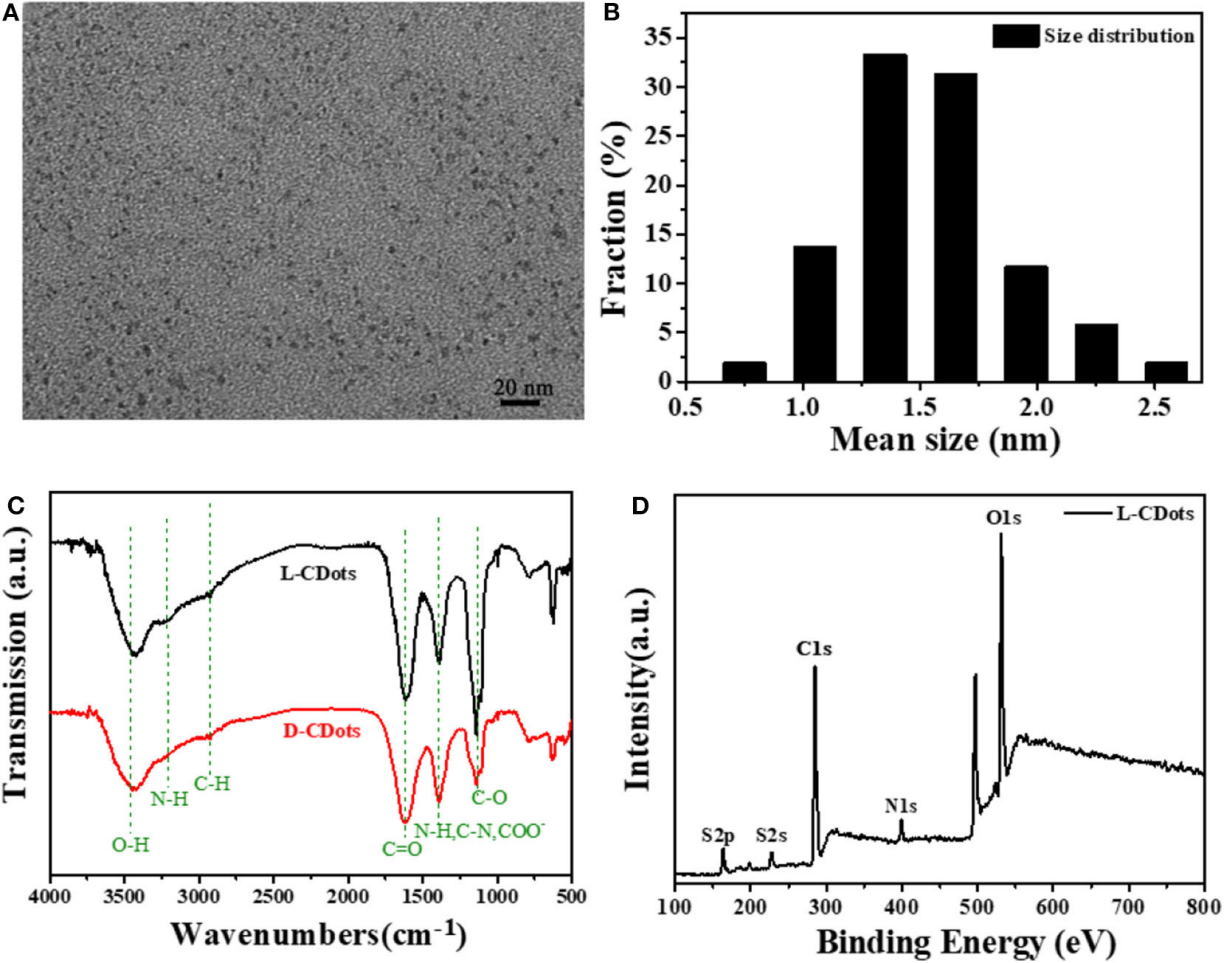
Morphological and structural characterization of L/D-Cys-CDots. **(A)** TEM images of L-Cys-CDots. **(B)** The corresponding size histogram of the L-Cys-CDots. **(C)** FT-IR spectrum of L/D-Cys-CDots. **(D)** XPS survey spectrum of L-Cys-CDots showing the O1s, N1s, C1s, and S2p.

Through the comparison of characterization results, the L-CDots and D-CDots are very similar in structure and performance. Therefore, this manuscript focuses on the introduce of L-CDots, more details of D-CDots are showed in Supplementary Materials. As shown in Figures 2A,B, the TEM image demonstrates that as-prepared L-CDots are well-dispersed with average size of 1.25 ± 0.5 nm in diameter. As shown in Figure 2C, the FT-IR spectra were compared with the L/D-CDots to employ for analyzing the surface functional groups, such as -COOH and -NH_2_. The infrared spectra of L/D-CDots are similar. The wide peak at 3,475 cm^−1^ and a small hump at 3,218 cm^−1^ are attributed to -OH vibration and N-H vibration. The peak at 2,970 cm^−1^ is assigned to C-H group. Two peaks centered at 1,700 cm^−1^ and 1,125 cm^−1^ correspond to the stretching vibrations of C=O and C-O (Mewada et al., 2013). The peak at 1,380 cm^−1^ represented C-N, N-H, and COO- bonds (Jiang et al., 2012).

To further analyze the chemical structure of L/D-CDots, the full-scan X-ray photoelectron spectroscopy (XPS) spectrum of L-CDots is depicted in Figure 2D, where S 2p, C 1s, N 1s, and O 1s correspond to the present four peaks located at 164.7, 286.05, 400.1, and 532.85 eV. The high resolution scan of the C 1s XPS spectrum of L-CDots, which is shown in Figure S2A can be divided into five component peaks, which correspond to C–C (284.6 eV), C-S (284.7 eV), C–N (sp3, 285 eV), C–O (sp3, 286.1 eV), and C=O (sp2, 288 eV) bonds (Qu et al., 2013). The expanded image of the O1s showed in Figure S2B. with three peaks at 531.2, 532.5, and 535.6 eV, which fitted with C-O, and C=O/N=O groups (Dong et al., 2014). As shown in Figure S2C, the N1S could be resolved into three peaks at 398.9, 400, and 400.8 eV, which are ascribed to graphitic N, pyridinic N and pyrrolic N (Qu et al., 2016). Figure S2D shows the partial XPS spectrum of S with two peaks at 163.3 and 164.3 eV, which can be identified as S 2p3/2 and S 2p1/2 (Cho et al., 2010).

**Figure 3:**
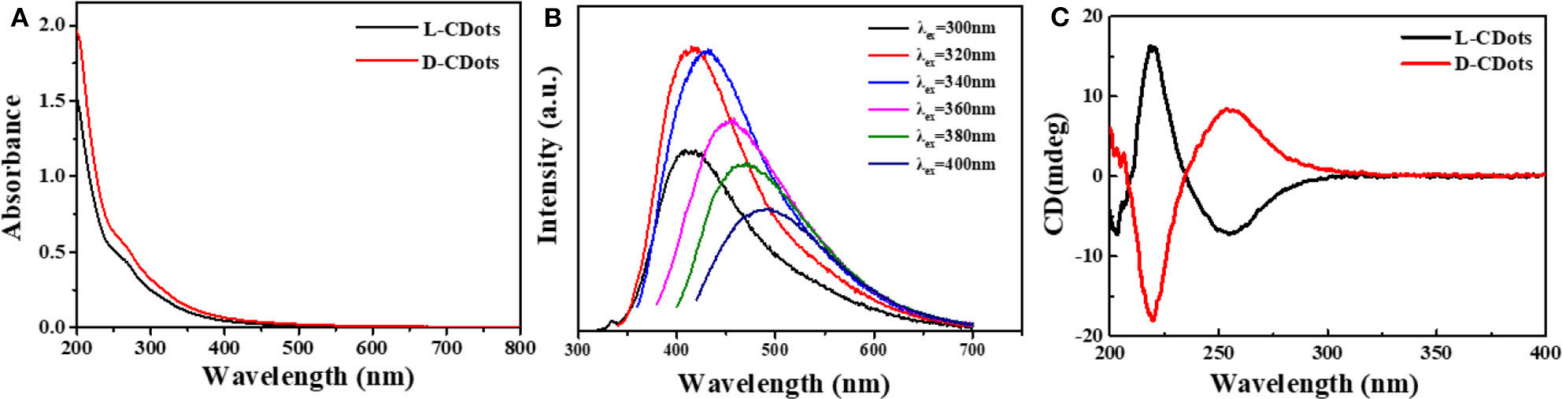
Photophysical and (chiro)optical characterization of L/D-CDots. Experiments performed in water at 298 K. **(A)** UV–Vis spectra of L-CDots (black line) and D-CDots (red line). **(B)** FL emission spectra (at different excitation wavelengths) of L-CDots (same emission profiles and intensities were observed for D-CDots). **(C)** CD spectra of L-CDots (black line) and D-CDots (red line).

As shown in Figure 3A, the UV-Vis absorption spectra of L- and D-CDots show that the absorption of L-CDots is similar to that of D-CDots. They have two distinct peaks at 265 and 320 nm, which are attributed to electron transitions from π-π^*^ (Yao et al., 2018) of C=C and C=O of the sp2-hybridized carbon network (Suzuki et al., 2016). The photoluminescence spectra of L- and D-CDots show that the optimal excitation and emission wavelengths are at 320 and 410 nm, respectively, and bright yellow luminescence is shown (Figure S3). The FL spectra of L-CDots at different excitation wavelength from 300 to 400 nm are shown in Figure 3B and the same emission profiles were observed in D-CDots (Figure S4), indicating that the L- and D-CDots exhibit similar optical properties. With the increase of excitation wavelength, the emission spectrum presents a red shift similar to that reported in literature (Li et al., 2011). Circular dichroism (CD) spectroscopy was also used to characterize the chirality of L-/D-dots. As shown in Figure 3C, the symmetric CD spectra of L- and D-CDots indicate that the successful synthesis of two types of CDots with opposite chirality. The CD spectrum of L-CDots presents one positive Cotton effects and one negative Cotton effects at 220 and 260 nm, respectively, in which the positive peak at 220 nm is related to the successful conferring signal of chirality from L-Cys (Figure S5). Another negative CD peak could be ascribed to the change from the π-π^*^ conjugation of the sp2-hybridized carbon network in L-CDots or the interaction of the carbon skeleton of L-Cys (Qu et al., 2013).

As a π electronic conjugate system, carbon dots have good electron transfer capability to realize charge transfer when combined with photoelectron donor (Cadranel et al., 2018). Whether a system realize electronic transfer is easy to realize chiral transfer has always aroused people's interest. Chiral transfer means the chirality of chiral materials, such as CDots, can be further transferred and amplified (Arcudi et al., 2017). Porphyrins are a kind of macromolecular heterocyclic compounds formed by the interconnection of α-carbon atoms of four pyrrole subunits through the methylene bridge (=CH−) (Wang et al., 2011). Due to their special chemical structure and electrical properties, supramolecular assembly can be carried out under certain conditions (Cao et al., 2017). In order to investigate whether it is possible to transfer the chiral signals from L-CDots and D-CDots to other achiral molecules, we discussed their non-covalent interactions with porphyrins.

**Figure 4:**
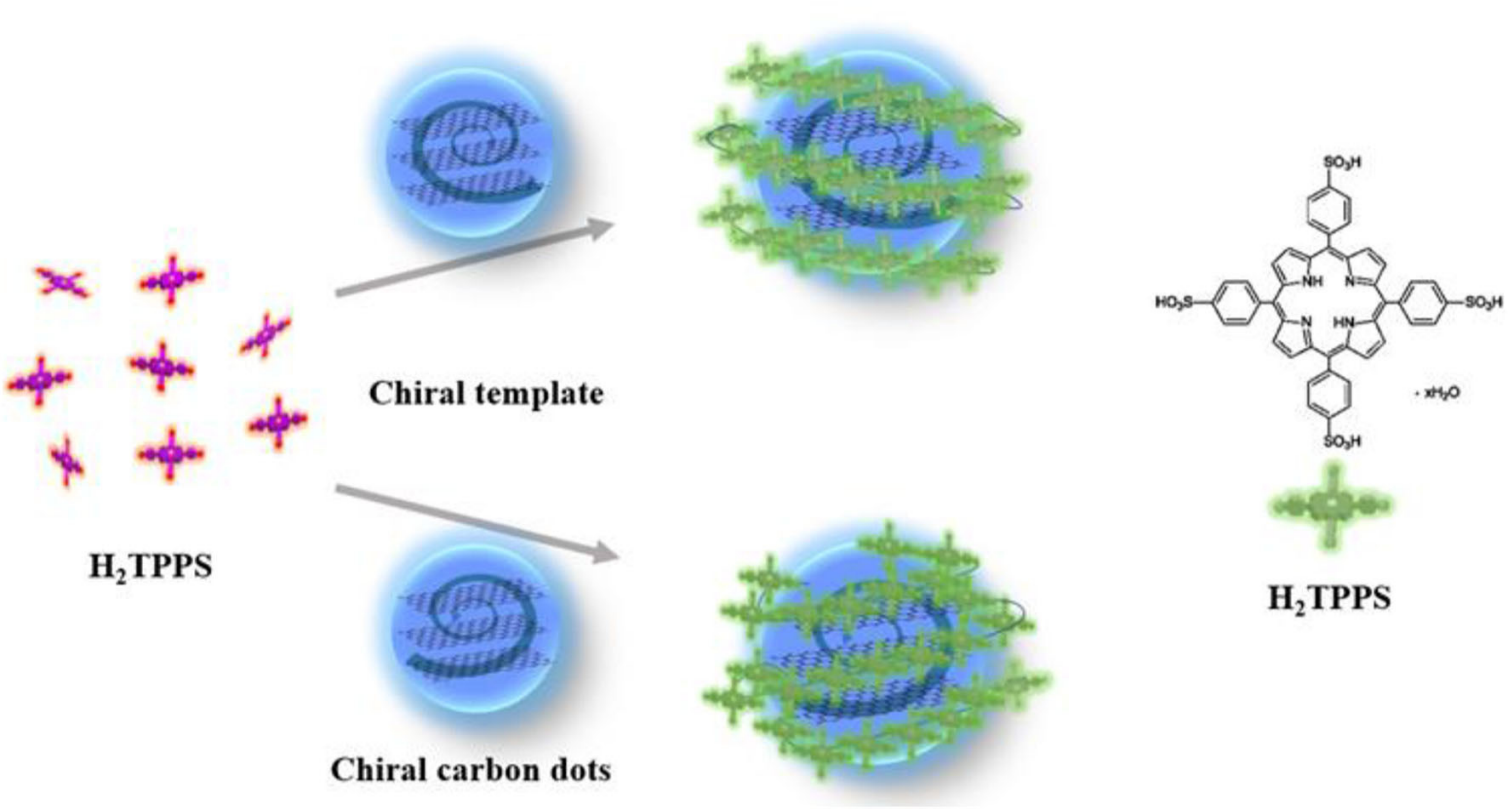
Schematic representation of L-Cys-CDots acting as chiral template after pH decrease trigger (purple = free base porphyrin, green = protonated porphyrin).

As shown in Figure 4, chiral carbon dots as templates for efficient formation of J-aggregated porphyrin were prepared. The anionic porphyrin, the 5,10,15,20-tetrakis(4-sulfonatophenyl)-porphyrin (H_2_TPPS) has the ability to form highly ordered J-(edge-to-edge) and H-(face-to-face) aggregates at very low pH or in the presence of various inorganic and organic cations was chosen (Ðorđević et al., 2018). Under specific experimental conditions (pH, ionic strength, concentration, etc.), protonated H_2_TPPS self-assemble into H- and J-aggregates of different shapes and sizes (Randazzo et al., 2018).

**Figure 5:**
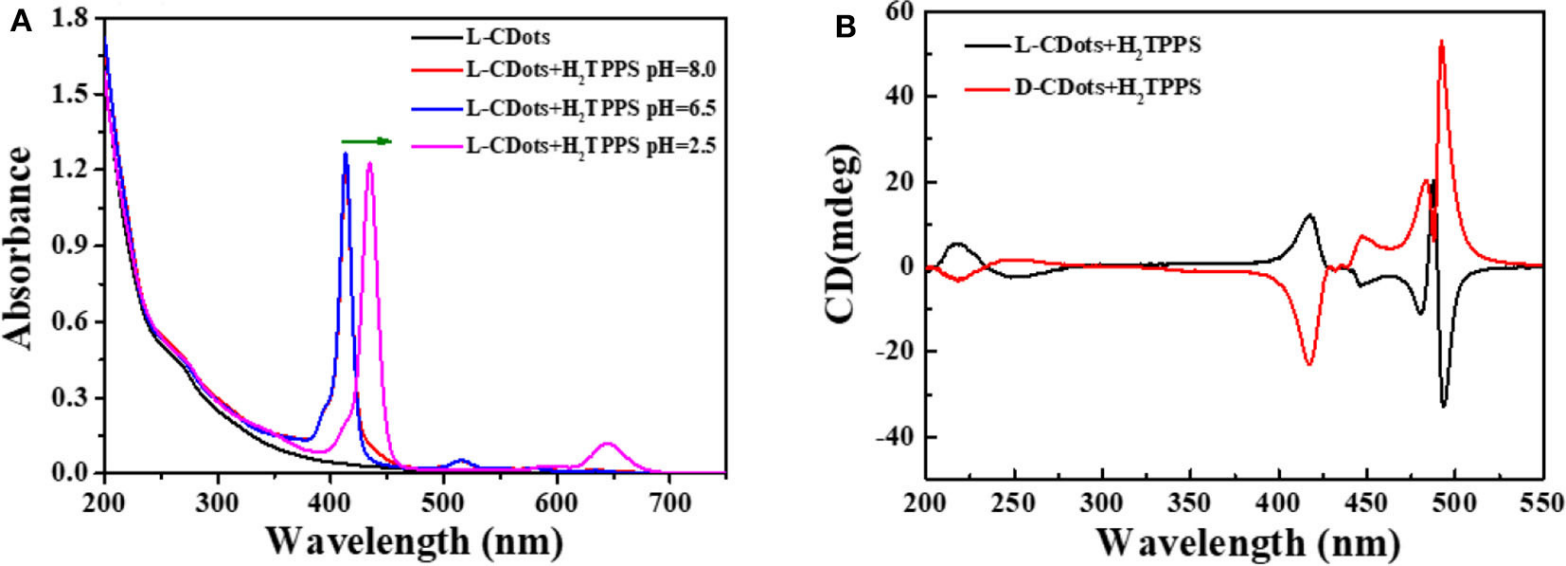
**(A)** UV–Vis spectra of L-CDots (black line) and L-CDots+H_2_TPPS solutions at different pH (H_2_TPPS concentration = 10 μM). **(B)** CD spectra of L-CDots (Black line) and D-CDots (Red line), both in the presence of H_2_TPPS (100 μM) at pH = 2.5.

As shown in Figure 5A, Figure S7B, the absorption spectra of L/D-CDots-H_2_TPPS present an intense absorption band at 433 nm (Soret band or B band) and weak absorption band at 590 nm (Q-bands) at pH = 8.0. Q (0,0) is the excitation from the lowest vibrational energy level of the singlet in the ground state to the lowest vibrational energy level of the first excited singlet, where Q (1,0) has a vibrational quantum in the first excited singlet. In the free-based porphyrin, Qx (0,0) and Qy (0,0) bands no longer degenerate due to the existence of proton axis, and there are four Q bands at the display (Spellane et al., 1980). Four weak visible bands between 520 and 650 nm are designated as Qy (1,0), Qy (0,0), Qx (1,0) and Qx (0,0) bands as the wavelength increases (Yang et al., 1999). The two distinct peaks at 265 and 320 nm are attributed to electron transitions from π−π^*^ of C=C and C=O of the sp2-hybridized carbon network of CDots. Because the surface of CDots is covered with amino and carboxyl groups, the surface charge of L/D-CDots can be adjusted by changing the pH value, which may regulate the interaction of CDots and porphyrins. In our research, the chiral carbon dots are negative at pH 8.0 (Figure S6). While when the pH was decreased from 8.0 to 6.5, the surface charge of CDots changes a lot, the color of L/D-CDots-H_2_TPPS turns red and the fluorescence is brighter under the UV light. The experimental results show that electrostatic interaction plays an important role in the interaction of CDots and porphyrins.

In this condition, porphyrins are still dominated by tetraanions and carbon dots' surface functional groups are protonated. To further promote the aggregation of H_2_TPPS, hydrochloric acid was used to decrease pH to 2.5 (Fleischer et al., 1971; Pasternack et al., 1972). Jelley (1936, 1937) and Scheiber (1936) discovered that an important feature of J-aggregate formation is that it will form a prominent, narrow, and strong red-shifted band. The emergence of the J-aggregate has attributed to the formation of monomer aggregates. The change in absorption spectrum is due to the reversible polymerization of the monomer into loosely bound polymers. Franck and Teller (1938) used Frankel's exciton theory (Frenkel, 1931a,b) to prove the point. The theoretical research on aggregate system is mainly focused on exciton coupling. Kasha combines the exciton and induced resonance excitation energy transfer theory of quantum mechanics (Kasha, 1963). Exciton model can give a correct description of excited states and local structure and sequence of aggregates at the nanoscale (Kasha, 1959; Higgins et al., 1996). The main step of the transition from monomer porphyrin to aggregate is the protonation of imino nitrogen on two pyrrole rings. Protonation induced the distortion of coplanar conformation from the near vertical direction of the middle aryl substituents to the average plane of the porphyrin macrocycle (Akins et al., 1996). The absorption spectrum shows that the two strong red-shifted narrow peaks at 440 nm (Soret band) and 650 nm (Q band) were appeared, which correspond to S0 → S2 and S0 → S1 transitions, respectively (Li X. et al., 2018). It proved the formation of J-type H_2_TPPS aggregates (Koti and Periasamy, 2003; Guo et al., 2011). There are very few examples of molecules forming pure J- or H-aggregates. The J-aggregate of H_2_TPPS is faster and more efficient than H-aggregate and the J-aggregate exhibits circular dichroism (CD) (Maiti et al., 1998). Under pH 2.5, new CD peak induced by porphyrin's aggregates were also observed. There are four signal CD peaks induced by L/D-CDots in the absorption region of porphyrin aggregates (Figure 5B) at 410, 440, 475, and 490 nm. We attribute the CD signals at 440, 475 and 490 nm to the chiral J-aggregate of H_2_TPPS (Purrello et al., 1998).

It is well-known that H_2_TPPS is achiral. As shown in Figure S7A, H_2_TPPS has no CD response in the absence of chiral compounds. However, the CD response in Soret region can be achieved by introducing appropriate chiral groups into the porphyrin ring. The chromogenic groups on the chiral side chain coupled with the porphyrin large conjugated ring, which made the conformation of the chiral group relatively stable. The amino and carboxyl groups on the surface of carbon dots were chiral groups, however they were completely deprotonized under pH 6.5. The electrostatic repulsion between negatively charged porphyrins and carbon dots increased at this condition, there the chiral transfer between carbon dots and porphyrin was failed. The formation of J-aggregates of H_2_TPPS requires the induction of organic or inorganic cations at low pH (Pasternack et al., 1972; Kishimoto et al., 1996). With the decrease of pH value, the formation of J-aggregate is promoted by the formation of cations by the amino protonation on the surface of CDots. As shown in Figure 5B, supramolecular porphyrin-CDots hybrids retains the CD signals of L/D-CDots and induces new chiral peaks at 400–500 nm. Compared with the pure L/D-CDots, the decrease of the signals at 200–275 nm may be due to the acidic environment. At 410 and 490 nm, the J-aggregates of H_2_TPPS exhibit strong CD signals, corresponding to the Soret band and J-band in the absorption spectrum (Koti and Periasamy, 2003). The band at 490 nm is intense and narrow. Electrostatic interactions are the driving forces of H_2_TPPS J-aggregate effectively inducing chirality.

As shown in Figure S8, the maximum anisotropy factor of circular polarization in absorption gabs of L-CDots-H_2_TPPS is −2.38 × 10^−3^ at λ = 496 nm and the gabs of D-CDots-H_2_TPPS is 1.7 × 10^−3^ at λ = 498 nm $({\text{g}}_{\text{abs }}=\;\frac{\text{2}\left(\epsilon_{\text{L}}-\epsilon_{\text{R}}\right)}{\epsilon_{\text{L}}\text{+ }\epsilon_{\text{R}}}=\frac{\Delta \epsilon }{\epsilon }\;=\;\frac{\text{ellipticity}}{\left(\text{32},\text{980 }\times \text{ A}\right)}$, ε is the extinction coefficient, where ε_L_ and ε_R_ represent left-handed and right-handed absorbance coefficients, respectively. Δε the difference of the extinction coefficients of left and right circularly polarized light, Δε = ε_L_-ε_R_. ellipticity and A was the chiral intensity of samples and the corresponding absorption). g_abs_ can be used to quantitatively estimate the single handed helical conformation of polymers. The larger g_abs_ value indicates that the excited state asymmetry of the system is stronger and the chiral transfer efficiency is higher. Compared with the g_abs_ value of D/L-CDots at λ = 220 nm (|1.19 × 10^−4^|), the g_abs_ value of D/L-CDots-H_2_TPPS is |4.4 × 10^−4^| at λ = 415 nm (Q-band), which proved the successful transfer of chirality from L/D-CDots to H_2_TPPS. The g_abs_ value at B-bands (|9 × 10^−4^| at 475 nm and |2 × 10^−3^| at 490 nm) is nearly eight and sixteen times higher than that at 220 nm, realizing chiral amplification. The g_abs_ values of the two are very close and have opposite signs, and have strong optical activity. The results show that the porphyrins are assembled in the chiral environment and chirality transfer and amplification are realized. Porphyrin aggregates borrow their chirality from the chiral template. Because of its remarkable kinetic inertia, they “memorize” the characteristics of the template and become chirality in essence (Lauceri and Purrello, 2005; Helmich et al., 2010).

**Figure 6:**
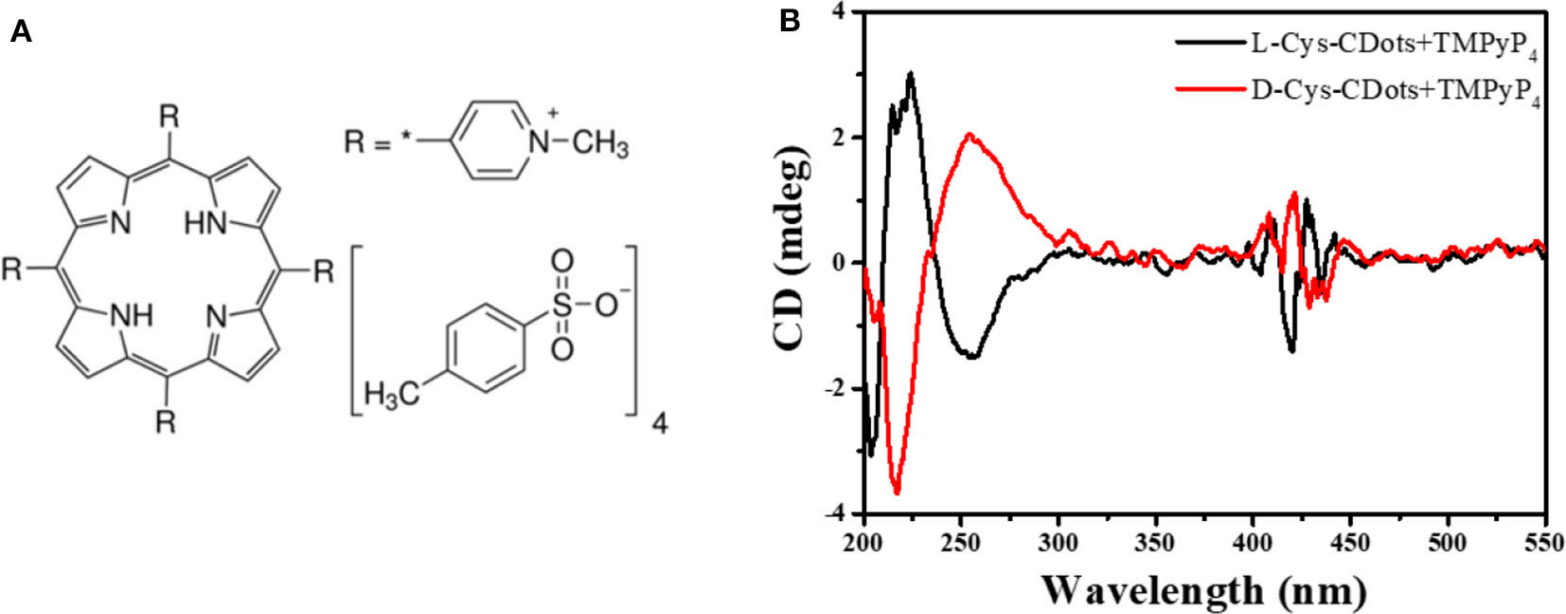
**(A)** Chemical structure of TMPyP_4_. **(B)** CD spectra of L-CDots (Black line) and D-CDots (Red line), both in the presence of TMPyP_4_ (100 μM) at pH = 7.0.

Another positive porphyrin, TMPyP_4_ [5, 10, 15, 20-tetrakis (1-methyl-4-pyridinio) porphyrin tetra(p-toluenesulfonate)] (Figure 6A), was also used to interact with chiral carbon dots at pH = 7.0 for 24 h. As shown in Figure 6B, three new absorption peaks appear at 400–430 nm. The absorption spectrum shows that the Soret band at 422 nm decreased and the Soret band exhibited a 5 nm bathochromic shift and a broader half-bandwidth compared with that of TMPyP_4_ alone, which proves electronic communication between the two delocalized π-electron systems of carbon dots and TMPyP_4_ (Figure S9B) (Jiang and Liu, 2004; Zhu and Liu, 2011). The symmetrical CD images and large bathochromic shift of the Soret band indicate that L/D-CDots and TMPyP_4_ have strong π-π stacking and electrostatic interactions.

Compared with Ðorđević's work, arginine was used as the core precursor and (R,R)- or (S,S)-1,2-cyclohexanediamine (CHDA) was used as the chiral source, the relatively high price of CHDA hinders the large-scale application of chiral CDots. While in our work, cysteine is the only precursor, acting as carbon source and chiral source. Cheap cysteine makes it possible for chiral CDots to be used on a large scale. Moreover, our CDots were prepared by hydrothermal method, making our CDots easier to prepare on a large scale. Secondly, our chiral CDots are rich in carboxyl and amino groups, which makes them excellent nanoparticles for assembling other molecules. In our system, electrostatic interactions between chiral groups around CDots and porphyrins are very important. Because of amino groups covered in the surface of CDots, the charge on CDots could be adjusted by reducing the pH value, thus affecting the interaction with anionic porphyrin. Under neutral condition, the carboxyl group on the surface of CDots will interact with the cationic porphyrin to initiate electron transfer, which realized the chirality transfer. Third, the assembly of H_2_TPPS aggregates in the two systems are different due to the different interfacial interactions. As shown in Figure 5B, under pH 2.5, new CD peaks compared to Ðorđević work induced by porphyrin's aggregates were observed. There are four signal CD peaks induced by L/D-CDots in the absorption region of porphyrin aggregates at 410, 440, 475, and 490 nm. Compared with the previous work, the intensity and shape of CD signal induced by J-aggregates strongly depend on the chiral template and a higher peak at 490 nm means more J-aggregates are formed in our work. It is shown that the size of chiral carbon dots and charge can affect the formation of the supramolecular chiral assembly process.

## Conclusion

Chiral carbon dots were prepared by one-step hydrothermal method from amino acids, which were used as templates to induce the chiral J-type aggregates of H_2_TPPS porphyrins under the condition of pH = 2.5 and TMPyP_4_ porphyrins under the condition of pH = 7.0. We have demonstrated that porphyrins can interact with carbon dots via electrostatic assembly to form supramolecular porphyrin-CDots hybrids, in which L/D-CDots were the chiral donors and porphyrins were the excited state chiral receptors. The chirality of carbon dots was transferred to porphyrin, and the chirality signal are amplified. Electrostatic interaction plays an important role in chiral transfer. Unfortunately, the binding equilibrium constant between cationic/anionic porphyrins and L/D-CDots cannot be obtained accurately. The production of chiral porphyrins will also promote the development of supramolecular chemical devices in biotechnology, such as chiral recognition and enantioselective catalysis. This work provides an example of using chiral carbon dots to modify the structure and function of porphyrins, a method that can be extended to other molecular or nanoscale blocks. Although chirality remains to be a huge challenge, especially for such carbon-based quantum dots, the research may trigger some advances in this field. Chiral carbon dots-porphyrins composites can be used in sensors that distinguish between different chiral substrates or be used in the preparation of chiral optical devices. The successful transmission of chiral information provides more options for the development of various chiral composite materials and the preservation of chiral information in the future.

## Data Availability Statement

All datasets presented in this study are included in the article/Supplementary Material.

## Author Contributions

XL and JL synthesized, fabricated and characterized materials, performed the data analysis, and prepared the manuscript draft. JC conceived the experiments and conducted the CD analyses. MZ revised the manuscript. YC and FX have designed the study and analyzed data. LF contributed to the conception of the study and approved the final version. All authors contributed to the article and approved the submitted version.

## Conflict of Interest

The authors declare that the research was conducted in the absence of any commercial or financial relationships that could be construed as a potential conflict of interest.
