# Supplementary material for: Chiral Self-Assembly of Porphyrins Induced by Chiral Carbon Dots
Source: Front Chem. 2020 Aug 7;8:670. doi: 10.3389/fchem.2020.00670 (PMC7427341; doi:10.3389/fchem.2020.00670)
Supplement: Supplementary file 1 [file Table_1.DOCX]

Supplementary Material

## Supplementary Figures


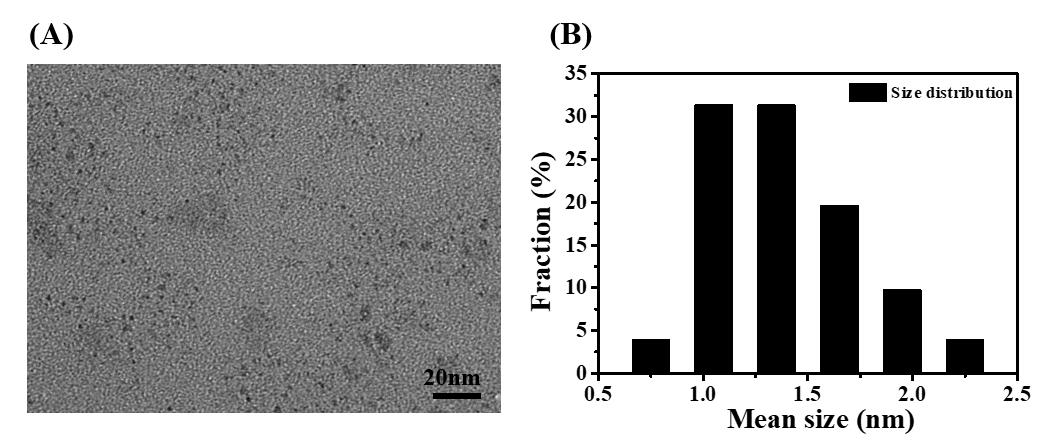


**Supplementary Figure 1.** (A) TEM images of D-Cys-CDots. (B) the corresponding size histogram of the D-Cys-CDots.


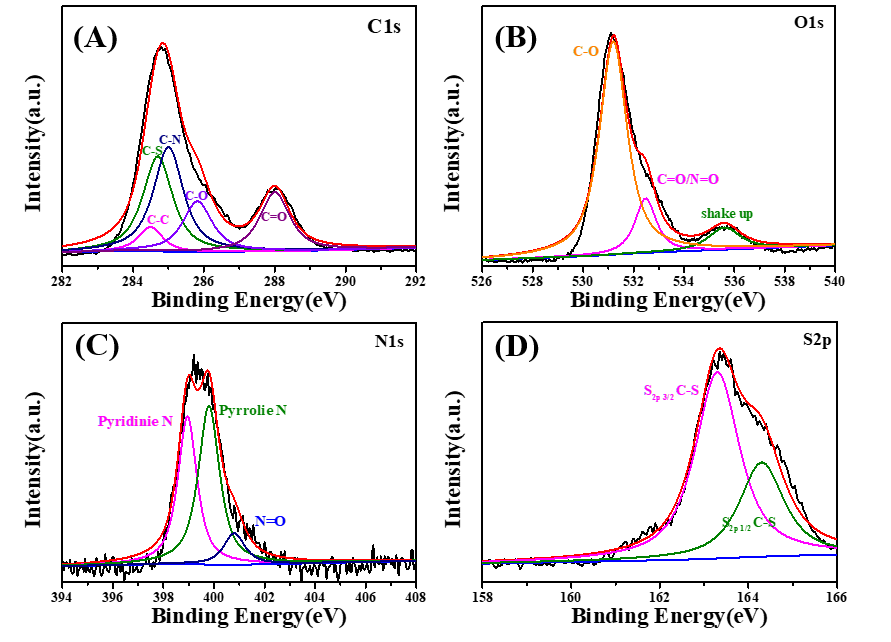


**Supplementary Figure 2.** High-resolution XPS spectra of (A) C 1s. (B) O 1s. (C) N 1s and (D)S 2p of L-Cys-CDots.


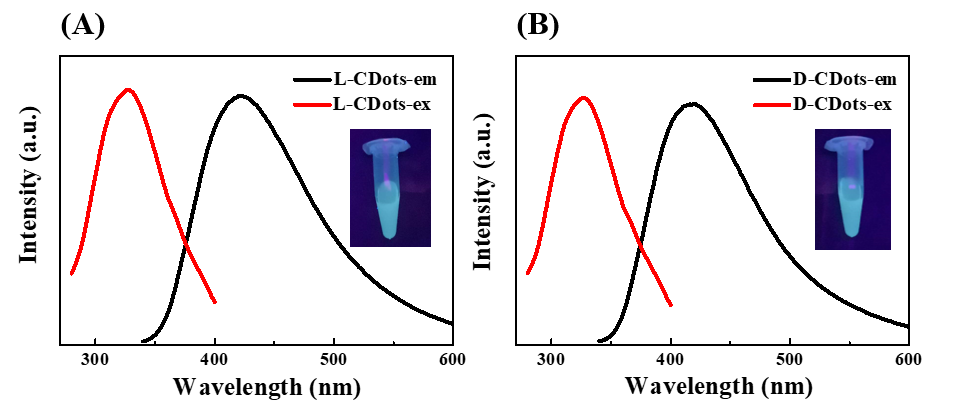


**Supplementary Figure 3.** PL excitation and emission spectra of (A) L-CDots. (B)D-CDots.


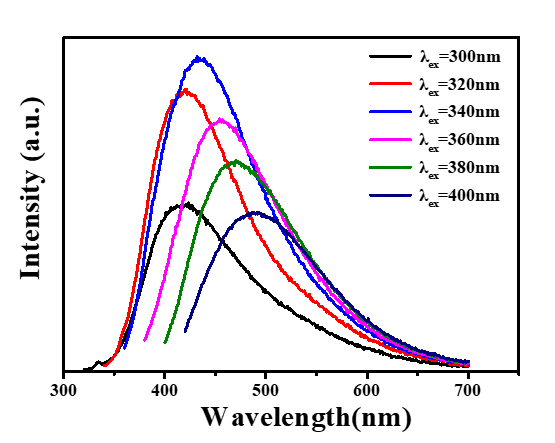


**Supplementary Figure 4.** FL emission spectra of D-CDots. Experiments performed in water at 298K.


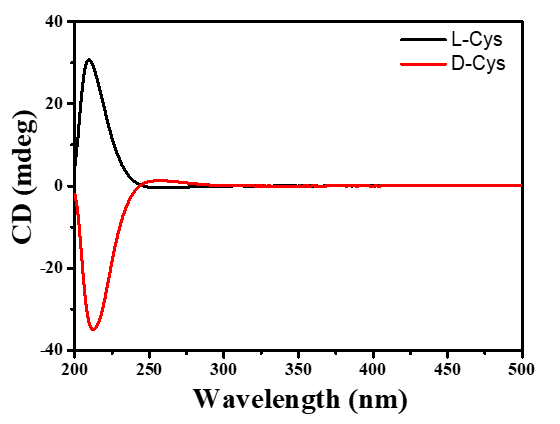


**Supplementary Figure 5.** CD spectra of L/D-Cys.


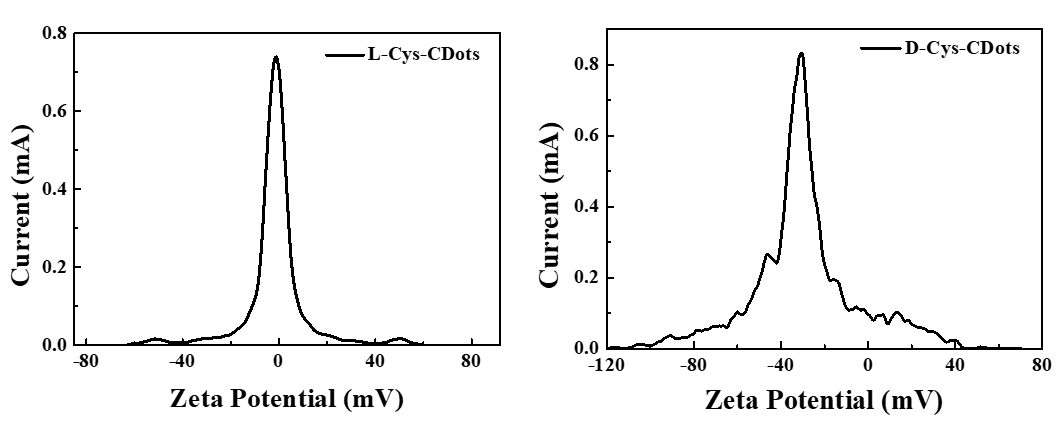


**Supplementary Figure 6.** Zeta potential of L/D-Cys-CDots.


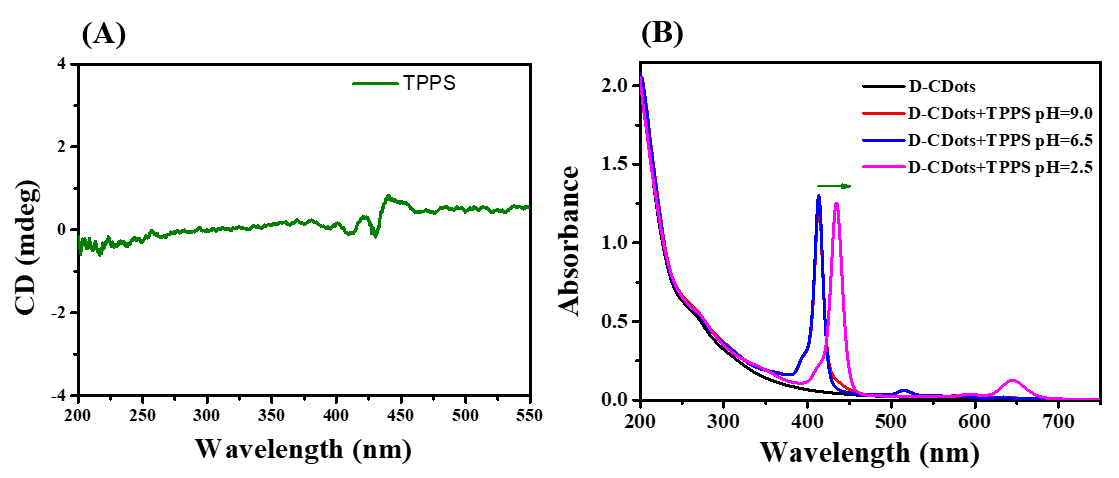


**Supplementary Figure 7.** (A) CD spectra of H_2_TPPS. (B) UV–Vis spectra of L-CDots (black line) and D-CDots + H_2_TPPS solutions at different pHs (H_2_TPPS concentration = 10μM).


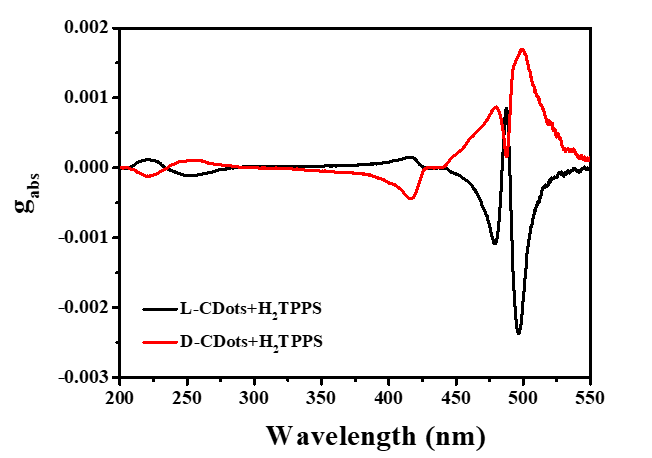


**Supplementary Figure 8.** The anisotropy factor of circular polarization in absorption gabs of L/D-CDots-H_2_TPPS.


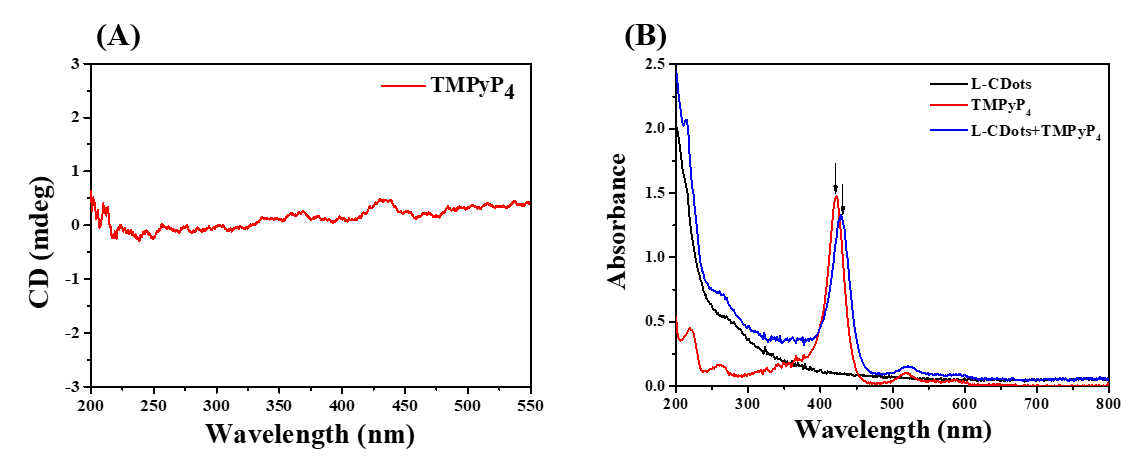


**Supplementary Figure 9.** (A) CD spectra of TMPyP_4_. (B) UV–Vis spectra of L-CDots (black line), TMPyP_4_(red line) and L-CDots + TMPyP_4_ (blue line) solutions at pH=7.0 (TMPyP_4_ concentration = 4μM).
